# AMPK attenuates SHH subgroup medulloblastoma growth and metastasis by inhibiting NF-κB activation

**DOI:** 10.1186/s13578-023-00963-2

**Published:** 2023-01-22

**Authors:** Jing Cai, Yue Wang, Xinfa Wang, Zihe Ai, Tianyuan Li, Xiaohong Pu, Xin Yang, Yixing Yao, Junping He, Steven Y. Cheng, Tingting Yu, Chen Liu, Shen Yue

**Affiliations:** 1 Department of Medical Genetics, Jiangsu Key Laboratory of Xenotransplantation, Nanjing Medical University, Nanjing, 211166 China; 2 Department of Neurosurgery, Children’s Hospital of Nanjing Medical University, Nanjing, 210093 China; 3 Departments of Pathology, Nanjing Drum Tower Hospital, The Affiliated Hospital of Nanjing University Medical School, Nanjing, 210008 China; 4 Department of Pathology, Suzhou Ninth People’s Hospital, Suzhou, 215200 China; 5 Jiangsu Key Lab of Cancer Biomarkers, Prevention and Treatment, Collaborative Innovation Center for Cancer Personalized Medicine, Nanjing Medical University, Nanjing, 211166 China

**Keywords:** AMPK, Shh signaling, Medulloblastoma, Gli1, NF-κB

## Abstract

**Background:**

Medulloblastoma (MB) is one of the most common malignant pediatric brain tumors. Metastasis and relapse are the leading causes of death in MB patients. The initiation of the SHH subgroup of MB (SHH-MB) is due to the aberrant activation of Sonic Hedgehog (Shh) signaling. However, the mechanisms for its metastasis are still unknown.

**Results:**

AMP-dependent protein kinase (AMPK) restrains the activation of Shh signaling pathway, thereby impeding the proliferation of SHH-MB cells. More importantly, AMPK also hinders the growth and metastasis of SHH-MB cells by regulating NF-κB signaling pathway. Furthermore, Vismodegib and TPCA-1, which block the Shh and NF-κB pathways, respectively, synergistically restrained the growth, migration, and invasion of SHH-MB cells.

**Conclusions:**

This work demonstrates that AMPK functions through two signaling pathways, SHH-GLI1 and NF-κB. AMPK-NF-κB axis is a potential target for molecular therapy of SHH-MB, and the combinational blockade of NF-κB and Shh pathways confers synergy for SHH-MB therapy.

**Supplementary Information:**

The online version contains supplementary material available at 10.1186/s13578-023-00963-2.

## Introduction

Medulloblastoma (MB) represents the most frequent central nervous system (CNS) malignant tumor in pediatrics and an embryonic tumor originating in the cerebellum [1, 2]. Four distinct molecular subgroups of MB termed Wingless (WNT), Sonic hedgehog (SHH), Group 3, and Group 4 were identified based on transcriptional profiling of large cohorts [3–5]. Relapses occur in 30% MB patients after surgery, and the metastasis rate and the 3 years survival rate of relapsed patients are 86% and 18% [6]. Metastatic cells detach from primary tumors and disseminate to the leptomeninges by passive spread through the cerebrospinal fluid or to the brain or other organs through a hematogenous route [7, 8]. Although the molecular mechanisms of MB primary tumors are well elucidated, the molecular defects underlying metastasis are still unclear.

SHH-MB arises from granule cell precursors in the developmental cerebellum following the aberrant activation of the Shh pathway [9]. Binding the secreted hedgehog ligand Shh to the membrane receptor Patched-1 (Ptch1) relieves the inhibitory effect of Ptch1 on the downstream G protein–coupled receptor Smoothened (Smo) and turns on the signaling events leading to the activation of transcription factor Gli1 [10]. Despite Smo inhibitor Vismodegib (GDC-0449), which FDA approves, having short-term effects for MB in clinical trials, drug resistance is frequently developed and eventually results in treatment failure [11–13]. Resistance mechanisms in MB may include mutations in drug-targeting molecules, new mutations downstream, and activation of other pathways in MB progress [14, 15]. To control the progress and relapse of MB, it is urgent to fully understand the specific metastasis mechanism of MB and find new molecular targets for that.

The AMP-activated protein kinase (AMPK), as an essential sensor of cellular energy status, can phosphorylate multiple substrates to respond to low energy levels and regulate cell metabolism and growth. AMPK is a heterotrimeric complex comprises catalytic α subunits (α1, α2), regulatory β (β1, β2) and γ subunits (γ1, γ2, γ3), which is activated by the phosphorylation of T172 site of AMPKα subunit [16]. AMPK has dual effects in different cancers, promoting and inhibiting tumor progress depending on its downstream substrates [17]. In Shh pathway, AMPK can interrupt GLI1 transcriptional activity through the phosphorylation of GLI1 at serines 102 and 408 and threonine 1074 [18, 19]. However, it is unknown whether the expression of AMPK subunits is abnormal in MB, and the mechanism of AMPK in the growth and metastasis of MB is not completely clear.

Here, we found that AMPK inhibited the proliferation and metastasis of SHH-MB cells by restraining the activation of Shh and NF-κB pathways. The combination of blocking NF-κB and Shh pathway had a synergically therapeutic effect on SHH-MB, revealing the AMPK-NF-κB axis as a potential target for SHH-MB treatment.

## Results

### Expression of PRKAA1 in MB is lower than in the normal cerebellum

**Fig. 1:**
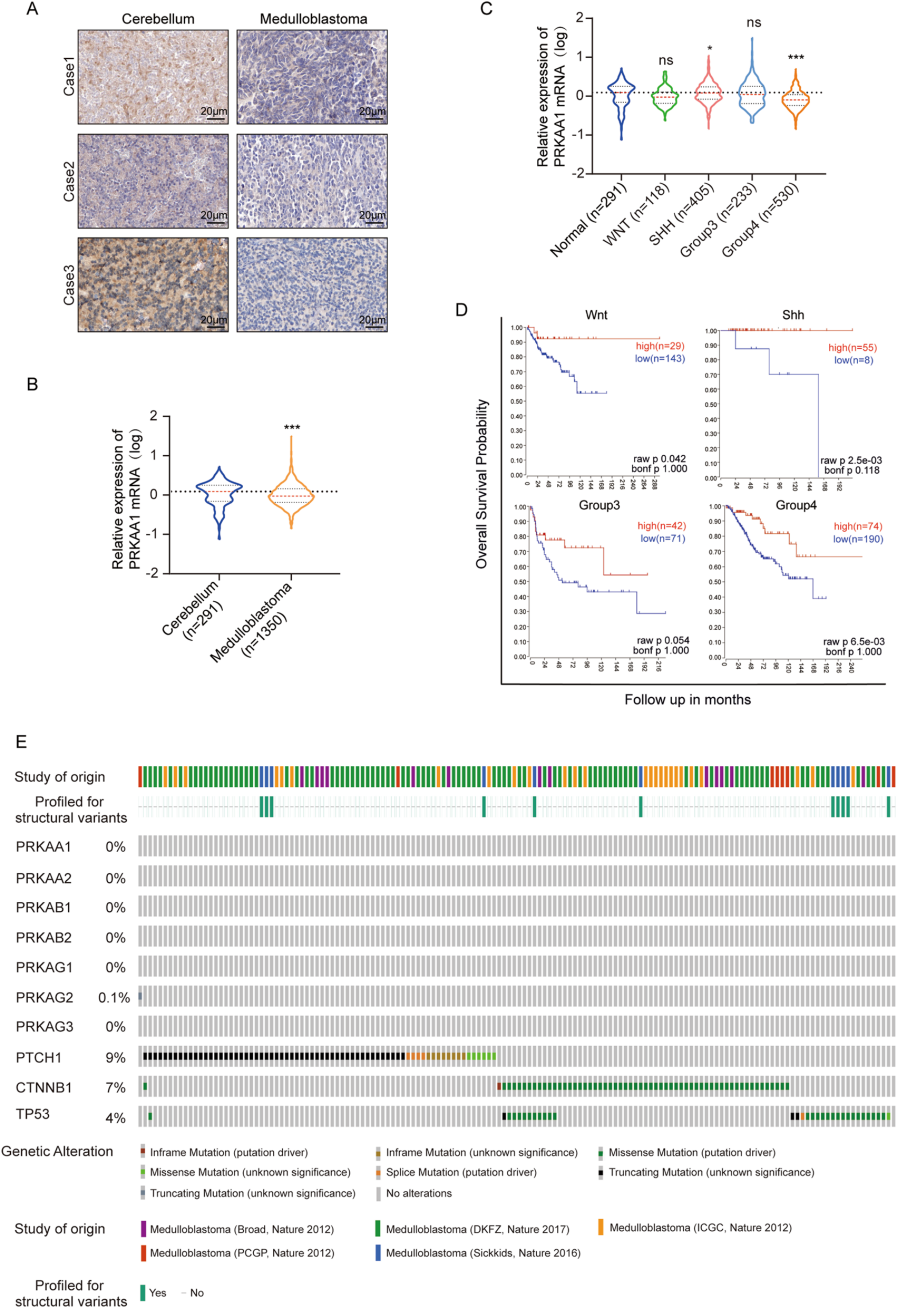
The abundance of AMPK expression was positively correlated with the prognosis of MB. **A** Representative immunohistochemical staining of phosphorylated AMPK in normal cerebellar tissues (n = 3) and medulloblastoma tissues (n = 3). Scale bar, 20 μm. **B** Comparison of mRNA abundance of *PRKAA1* in normal cerebella (n = 291) and medulloblastoma (n = 1350). Data from GSE124814. Comparison of two groups, ***P < 0.001. **C** Expression of *PRKAA1* in four subgroups of MB samples from GSE124814 dataset. Comparison to normal, *P < 0.05, ***P < 0.001. **D** Kaplan–Meier analysis of *PRKAA1* in different subtypes of medulloblastoma. Data from GSE85217. **E** TCGA figure showing mutations in selected genes. The figure panel was created using the cBioPortal for the MB data set available on the portal

To identify the connection between AMPK and MB progression, we first performed immuno-histochemical staining of normal cerebellum and MB tissues. 2 in 5 SHH-MBs and 5 in 6 Group 4 MBs showed significantly reduced signal of phosphorylated AMPKα compared to normal cerebellum (Fig. 1A). The differential mRNA expression analysis between MB and normal cerebellum using the MB patient dataset (GSE124814) found that *PRKAA1* (AMPKα1) mRNA expression was down-regulated in MBs (Fig. 1B). These results suggest that activated AMPK is reduced in MB tissues. *PRKAA1* mRNA expression was not consistent in four distinct molecular subgroups (WNT, SHH, Group 3, and Group 4), which was significantly decreased in Group 4 MB tissues, but slightly increased in SHH-MB compared to normal (Fig. 1C). Kaplan-Meier survival analysis showed that the patients with higher *PRKAA1* expression had longer survival durations than those with lower *PRKAA1* expression (Fig. 1D). The positive correlation between *PKRAA1* levels and survival time was shown in all four subgroups of MB patients. These results suggest that high-level expression of *PRKAA1* may be a sign of favorable prognosis for MB patients.

It was noted that there was no mutation in several genes denoting AMPK subunits reported in MB tissue from The Cancer Genome Atlas (TCGA) cohort, although *CTNNB1*, *PTCH1*, and *TP53* genes were missense-mutated or deleted in 5–10% cases (Fig. 1E). It suggested that repressed transcription and activation of AMPKα in MB tissues were likely due to epigenetic aberrations.

### AMPK suppresses DAOY cell growth

Since the SHH subtype of MB has an ostensibly simple tumor origin and is well-studied, the SHH subtype of MB cell line DAOY was selected for our further study. To determine whether AMPKα controls MB cells’ growth and malignant progression, we used small interfering RNA (siRNA) to knock down *PRKAA1* expression in DAOY cells. The knockdown efficiency of siPRKAA1 was validated by Western Blot (Additional file 1: Fig S1A). Knockdown of *PRKAA1* promoted the proliferation of DAOY cells according to CCK-8 assay (Additional file 1: Fig S1B) and EdU incorporation assay (Additional file 1: Fig S1C, D). Besides, siRNA targeting *PRKAA1* encouraged DAOY cells to form colonies (Additional file 1: Fig S1E, F). In addition, flow cytometric analysis revealed that siRNA against *PRKAA1* significantly increased the proportion of cells in the S-phase and decreased the proportion of cells in the G1-phase (Additional file 1: Fig S1G, H). Moreover, according to the CCK-8 assay, knockdown of *PRKAA1* also promoted the proliferation of D283 Med, a representative Group 3/4 subgroup MB cell line (Additional file 1: Fig S1I, J).

**Fig. 2:**
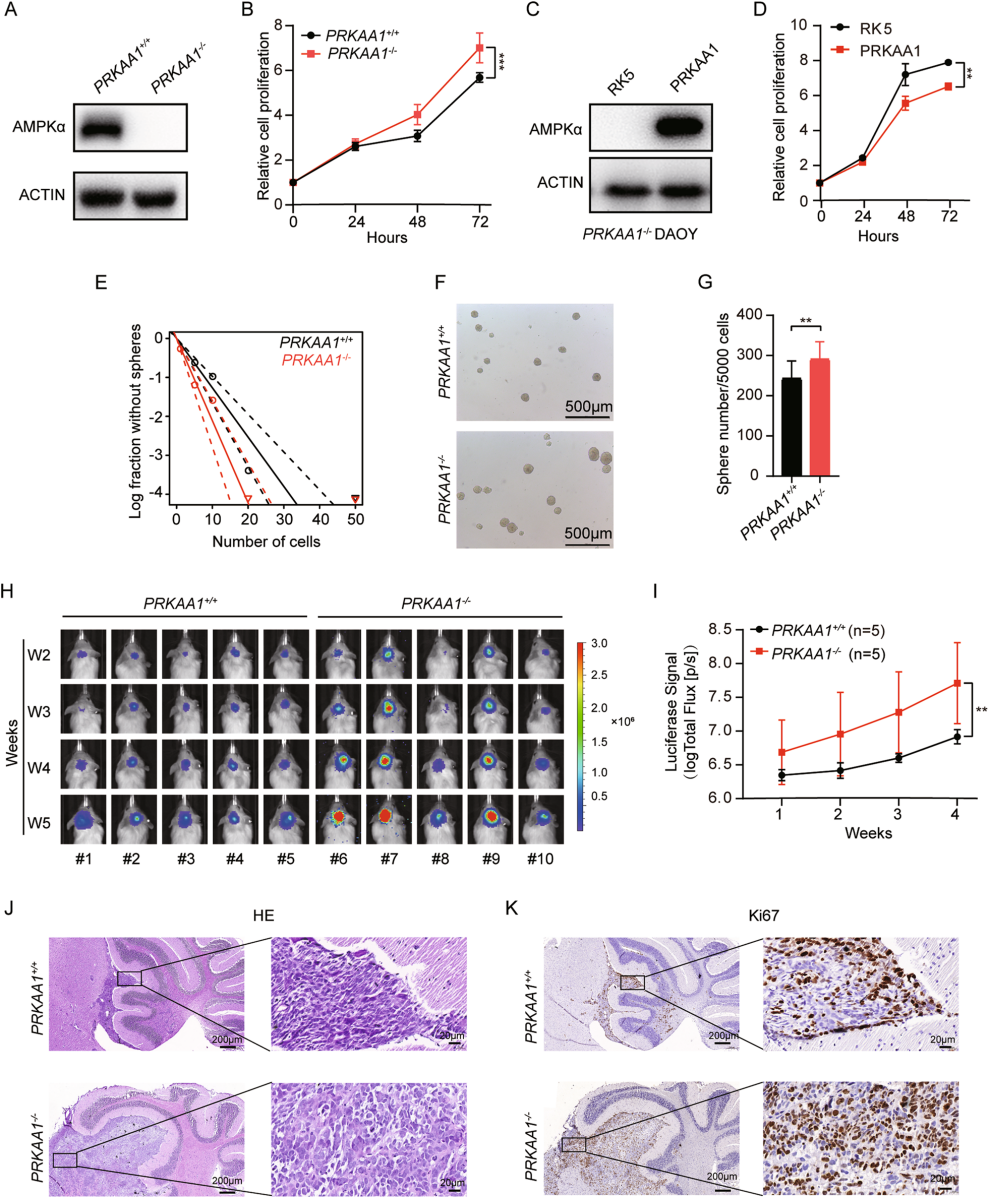
Knockout of AMPKα promotes DAOY proliferation. **A** Western blot detection of AMPKα protein level in *PRKAA1*^+/+^ and *PRKAA1*^−/−^ DAOYs. **B** Proliferation of *PRKAA1*^+/+^ and *PRKAA1*^−/−^ DAOYs were assessed by CCK-8. *** P < 0.001. **C** Western blot detection of AMPKα protein level in *PRKAA1*^−/−^ DAOYs transfected with *PRKAA1* cDNA. **D** Cell viability of transfected *PRKAA1*^−/−^ DAOYs was detected by CCK-8. ** P < 0.01. **E** Tumorsphere formation ability was detected by extreme limiting dilution assays. **F** Representative images of tumorspheres formed by *PRKAA1*^+/+^ and *PRKAA1*.^−/−^ DAOYs. **G** Quantification of the number of spheres (per 5000 cells) formed by DAOYs. Data in bar graphs are presented as mean ± SEM from three independent experiments. ** P < 0.01. **H** The time course of in vivo fluorescence images of NOD-SCID mice implanted with DAOY-Luc cells in the cerebellum using IVIS Spectrum In Vivo Imaging System. **I** Quantification of total Flux of mice. ** P < 0.01. **J** HE staining and **K** IHC staining of Ki67 of cerebellar tumors generated by implanting DAOY-Luc cells (5 × and 40x)

Next, to eliminate the off-target effects of siRNA, we used CRISPR/Cas9 genome editing technology to generate *PRKAA1*^*−/−*^ DAOY cells. Two short guide RNAs were designed to target sites at exons 8 and 10 of *PRKAA1* gene, and a 2062 bp deletion was induced in the DAOY genome (Additional file 2: Fig S2A). The knockout of *PRKAA1* was verified by Sanger sequencing and Western blot (Additional file 2: Fig S2A, Fig. 2A). The proliferation ability of *PRKAA1*^*−/−*^ DAOY cells was enhanced compared with *PRKAA1*^+*/*+^ cells shown by the CCK-8 assay (Fig. 2B). Moreover, the introduction of exogenous *PRKAA1* gene compromised the proliferation ability of *PRKAA1*^*−/−*^ DAOY cells (Fig. 2C, D). These results indicated that AMPK controls the proliferation of DAOY cells.

DAOY cells could form tumorsphere in 3D culture, suggesting their characteristics of cancer stem cells. The self-renewal capability was increased, as assessed by extreme limiting dilution assays, following the deletion of *PRKAA1* in DAOY cells (Fig. 2E). *PRKAA1*^*−/−*^ cells generated higher numbers of tumorspheres than *PRKAA1*^+*/*+^ cells (Fig. 2F, G). In brief, we found that AMPK limits the self-renewal ability of DAOY cells.

To determine the contribution of AMPK to tumor growth in vivo, we monitored the growth of *PRKAA1*^*−/−*^ DAOY cells in the cerebellum of NOD-SCID mice. *PRKAA1*^*−/−*^ cells and their *PRKAA1*^+*/*+^ control were infected with lentiviral particles to express Firefly Luciferase (Luc) gene stably and subsequently referred to as *PRKAA1*^*−/−*^-Luc and *PRKAA1*^+*/*+^-Luc cells. These cells were then implanted into the cerebella of NOD-SCID mice. Animals were subjected to weekly in vivo bioluminescent imaging (BLI) to monitor the orthotropic tumor growth (Fig. 2H). Serial BLI images showed that xenografts from *PRKAA1*^*−/−*^-Luc cells grew faster than those from control cells (Fig. 2I). Then the animals were sacrificed, and the cerebellum tissues were collected for histological analysis. HE staining of cerebellum tissues confirmed the presence of xenografts in the cerebellum (Fig. 2J). *PRKAA1*^*−/−*^ MB lesions had more Ki67-positive cells, indicating enhanced proliferation ability (Fig. 2K). Subcutaneous xenografts in nude mice provided additional evidence for the growth-suppressing effect of AMPK (Additional file 2: Fig S2B). *PRKAA1*^*−/−*^ xenografts exhibited significantly increased tumor volumes, weights, and the percentage of Ki67-positive cells (Additional file 2: Fig S2C-E). Taken together, these results suggest that AMPK could hamper the growth of SHH subgroup MB cells.

### AMPK controls DAOY cell migration and invasion

**Fig. 3:**
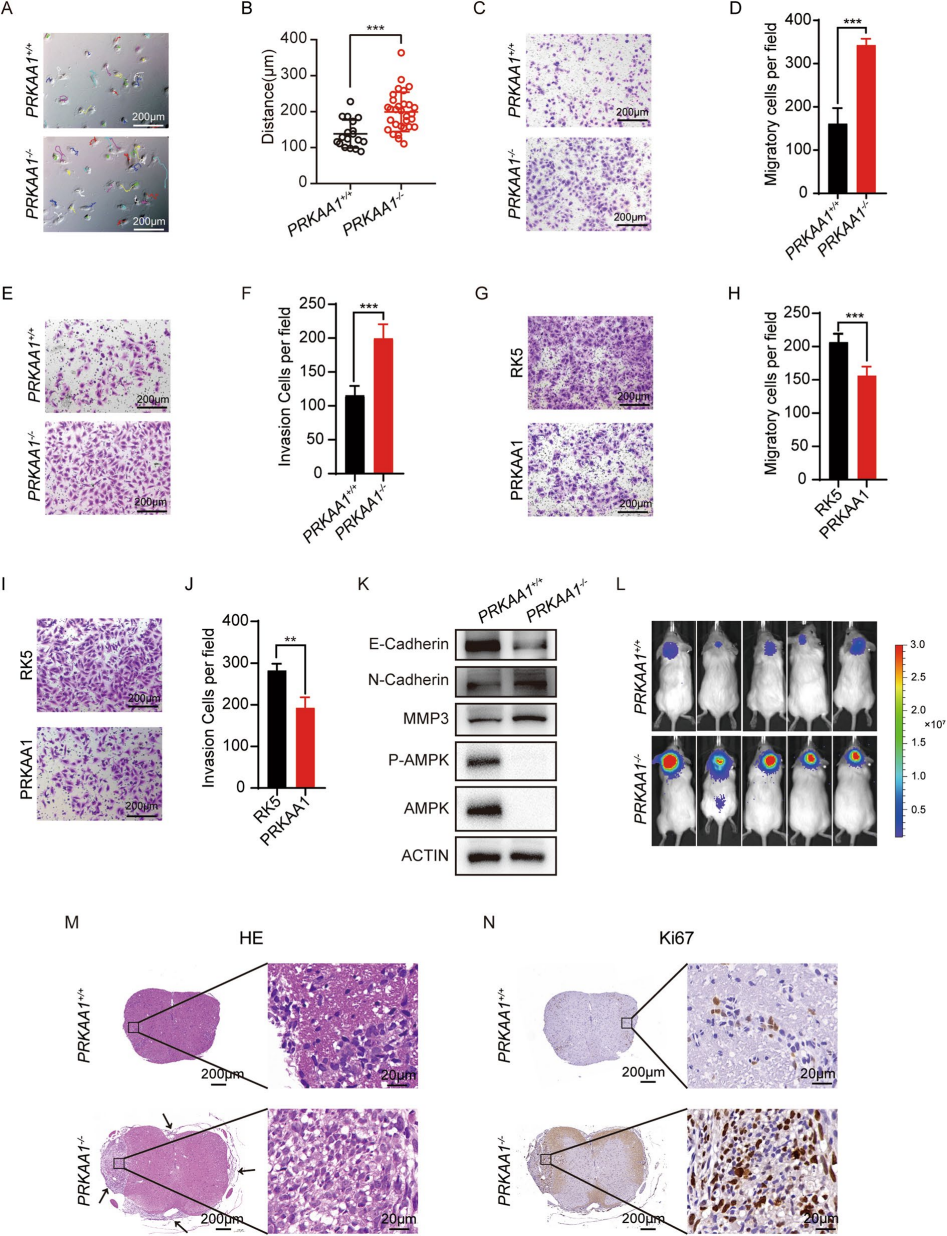
Knockout of AMPKα promotes DAOY migration and invasion. **A**-**B** Trajectory of DAOYs were recorded by Celldiscoverer 7 (Zeiss) and ImageJ. *** P < 0.001. **C** Transwell migration assay of knockout cells. **D** Quantification data performed the average migration ± SEM from three independent experiments. *** P < 0.001. **E** Transwell invasion assay of knockout cells with matrigel. **F** Quantification data performed the average invasion ± SEM from three independent experiments. *** P < 0.001. **G** and **I** Evaluation of migration and invasion ability of *PRKAA1*^−/−^ DAOYs transfected with *PRKAA1* plasmids by transwell assays with and without matrigel. **H** and **J** Quantitative data from three independent experiments performed as in G and I. ** P < 0.01. **K** Western blot detection of AMPKα, P- AMPKα and EMT markers in *PRKAA1*^+/+^ and *PRKAA1*.^−/−^ DAOYs. **L** In vivo fluorescence images of NOD-SCID mice implanted with DAOY-Luc cells at Week7. **M** HE staining and **N** IHC staining of Ki67 of spinal cord from NOD-SCID mice implanted with DAOY-Luc cells (5 × and 40x)

We noticed that introduction of *siPRKAA1* changed the morphology of DAOY cells, in addition to their proliferation capacity. The DAOY cells carrying *siPRKAA1* showed an elongated spindle shape rather than the polygonal shape of the control DAOY cells carrying *siNC* in culture in vitro (Additional file 3: Fig S3A). By Phalloidin labeling, we found that the control cells showed diffusely distributed actin filaments (F-Actin) and filopodia-like protrusions. In contrast, *PRKAA1* knockdown cells displayed significant aggregation of F-actin bundles and smooth lamellipodia-like edges (Additional file 3: Fig S3A). Furthermore, we performed transwell assays to determine the migratory and invasive capacities of DAOY cells. Knockdown of *PRKAA1* significantly enhanced the migratory and invasive abilities of DAOY cells (Additional file 3: Fig S3B-E). Statistical analysis of tracked cells indicated that the average movement distance of *PRKAA1*^*−/−*^ cells was longer than *PRKAA1*^+*/*+^ cells (Fig. 3A, B), suggesting that the knockout cells displayed increased cell motility. Moreover, *PRKAA1*^*−/−*^ cells showed enhanced migration and invasion abilities in transwell assays (Fig. 3C-F), and rescue of the *PRKAA1*^*−/−*^ cells with *PRKAA1* reversed these effects (Fig. 3G-J). Meanwhile, the migration capability of D283 Med cells was enhanced following depletion of *PRKAA1* (Additional file 3: Fig S3F, G). These data suggest that AMPK controls the invasiveness and metastasis of MB cells.

AMPK plays an important role in regulating the epithelial-mesenchymal transition (EMT). *PRKAA1*^*−/−*^ DAOY cells and their subcutaneous xenografts showed the decreased expression of epithelial cell marker E-cadherin and increased expression of mesenchymal cell markers N-cadherin and MMP3 versus control *PRKAA1*^+*/*+^ cells (Fig. 3K and Additional file 3: Fig S3H). siPRKAA1 also promoted the EMT process by showing increased levels of mesenchymal cell markers (Additional file 3: Fig S3I). To determine whether *PRKAA1*^*−/−*^ DAOY cells exhibited increased metastatic potential in vivo, we separately transplanted *PRKAA1*^*−/−*^-Luc and *PRKAA1*^+*/*+^-Luc DAOY cells into mouse cerebella and monitored the leptomeningeal metastasis seven weeks after implanting. *PRKAA1*^*−/−*^ cells produced spinal metastases based on the bioluminescent imaging and pathological examination (Fig. 3L–N).

### AMPK-GLI1 inhibits DAOY cell growth but not metastasis

**Fig. 4:**
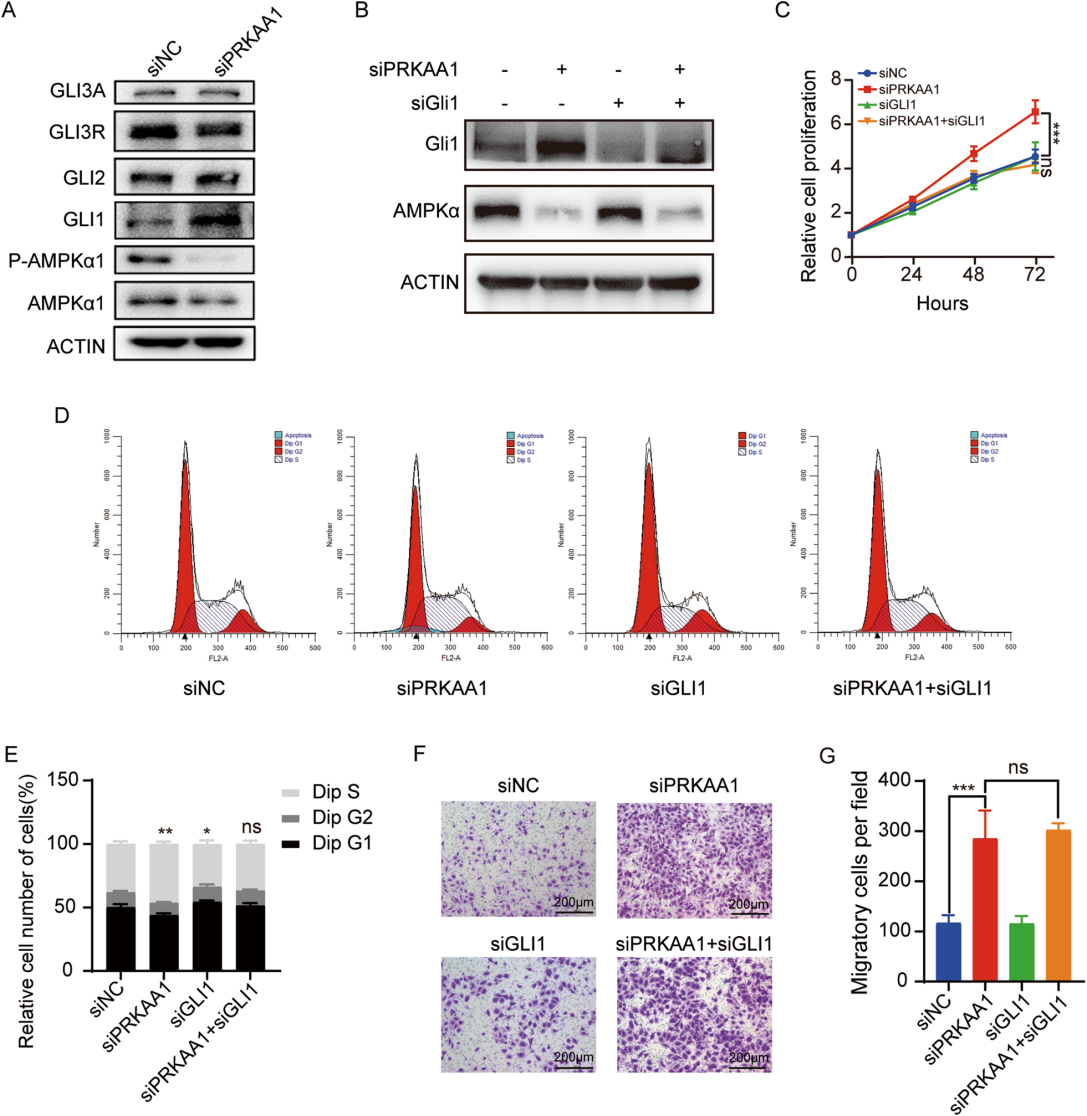
AMPKα inhibits the proliferation but not migration of DAOY through GLI1. **A** Western blot detection of AMPKα, P- AMPKα and GLI1 families in DAOYs transfected with *PRKAA1* siRNA or non-silencing control. **B** Western blot detection of AMPKα and GLI1 protein levels in DAOYs transfected with siPRKAA1 and siGLI1 alone or together. **C** Proliferation of DAOYs transfected with siRNAs were assessed by CCK-8. **D** Cell cycle of DAOYs transfected with *PRKAA1* or *GLI1* siRNA were detected by flow cytometry. **E** Quantification data of three independent experiments as in (**D**). **F** Transwell migration assay of DAOYs transfected with siRNAs and its quantification data (**G**) performed the average migration ± SEM from three independent experiments. *** P < 0.001

Although it was reported that activation of AMPK suppresses MB cell growth through inhibition of GLI1 activity and expression by phosphorylating and destabilizing GLI1 protein [20], the molecular mechanism by which AMPK inhibits DAOY cells proliferation and metastasis remains not entirely clear. Since SHH-MB is characterized by an overall deregulation of SHH pathway, we performed a western blot analysis to examine the levels of transcription factors GLI1-3 (Fig. 4A). As expected, GLI1 accumulated at a higher level *PRKAA1*-knockdown DAOY cells, While the levels of GLI2, GLI3 activator and repressor were not changed (Fig. 4A). To investigate whether the accumulated GLI1 contribute to the overgrowth and metastasis of DAOY cells driven by AMPKα loss, we performed a double-knockdown of both *PRKAA1* and *GLI1* in DAOY cells (Fig. 4B). Significantly, *GLI1* depletion abolished accelerated cell proliferation in *PRKAA1* knockdown cells, as illustrated by CCK-8 assay (Fig. 4C). The flow cytometric proliferation assay confirmed that *GLI1* depletion rescued *PRKAA1* deficiency-induced accumulation of cells in S-phase (Fig. 4D, E). These results demonstrated that the accumulation of GLI1 is responsible for the accelerated proliferation of DAOY cells seen upon *PRKAA1* loss, which is consistent with the literature reports. However, si*GLI1* did not counteract the enhanced migration of DAOY by si*PRKAA1* in the transwell migration assay (Fig. 4F, G), suggesting that AMPK hampered DAOY migration through some unknown signaling pathways except for the SHH-GLI1 pathway.

### AMPK restrains DAOY cell metastasis through inhibiting NF-κB pathway

**Fig. 5:**
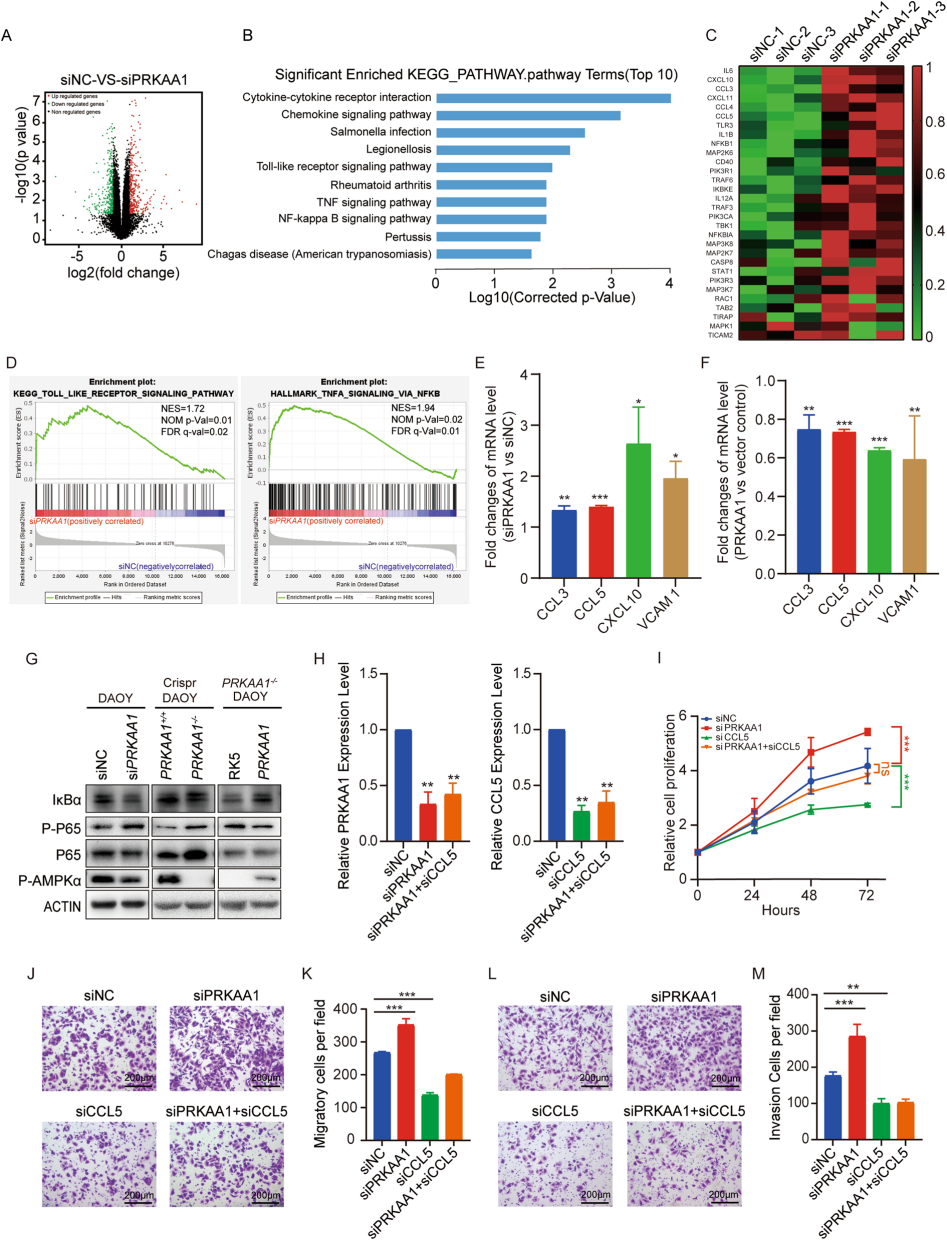
AMPKα represses the activation of NF-κB pathway. **A** Volcano plot showing the differential expression genes (DEGs) from DAOYs transfected with PRKAA1 siRNA or non-silencing control. **B** KEGG pathway enrichment of DEGs. **C** Heatmap of genes in Toll-like receptor signaling pathway. **D** GSEA enrichment plots of gene sets from Toll-like receptor signaling pathway and TNF-α signaling via NF-κB in the si*PRKAA1* group. **E** RT-PCR detection of *PRKAA1* and NF-κB target genes mRNAs in DAOYs transfected with *PRKAA1* siRNA. **F** RT-PCR detection of *PRKAA1* and NF-κB target genes in *PRKAA1*.^−/−^ DAOYs transfected with *PRKAA1*. **G** Western blot detection of phosphorylated AMPKα (P- AMPKα), IκBα, P65, and phosphorylated P65 (P-P65) in DAOYs. **H** RT-PCR detection of *PRKAA1* and *CCL5* mRNAs in DAOYs transfected with *PRKAA1* siRNA. **I** Cell proliferation of DAOYs transfected with *PRKAA1* or *CCL5* siRNA were assessed by CCK-8. Migration **J** and invasion **L** abilities of DAOYs transfected with *PRKAA1* or *CCL5* siRNA evaluated by transwell assays with and without matrigel. **K** and **M** are quantification data of three independent experiments as in (**J**) and (**L**). Data in bar graphs are performed as mean ± SEM. * P < 0.05, ** P < 0.01, *** P < 0.001

To further elucidate the underlying mechanism that AMPK inhibits the proliferation and metastasis of DAOY, we performed transcriptome sequencing in DAOY depletion of *PRKAA1*. Knockdown of *PRKAA1* induced significant gene expression changes in DAOY cells. The volcano plot depicted that 334 genes were up-regulated and 317 were down-regulated (Fig. 5A). The KEGG pathway analysis showed the enrichment for NF-κB pathway (Fig. 5B). The gene set enrichment analysis (GSEA) and a hierarchical clustering heatmap analysis also revealed a significant activation of NF-κB pathway in *PRKAA1* knockdown DAOY cells (Fig. 5C, D). The effects of AMPK depletion on activating NF-κB signal pathway transduction were validated by real-time qPCR of *CCL3, CCL5, CXCL10,* and *VCAM1*, the downstream target genes of the NF-κB pathway (Fig. 5E). Moreover, the introduction of exogenous *PRKAA1* gene reduced the transcripts of those target genes in *PRKAA1*^*−/−*^ cells (Fig. 5F). Activation of NF-κB pathway occurs after the degradation of pathway inhibitor IκBα and the phosphorylation of NF-κB p65. Western blots showed that the phosphorylation of P65 was increased in cells knockdown of *PRKAA1*, companying with the decrease of IκBα, which suggested the activation of the NF-κB signal pathway. Meanwhile, similar results existed in *PRKAA1*^*−/−*^ cells and were reversed by the introduction of exogenous *PRKAA1* cDNA (Fig. 5G). It is noticed that the total levels of P65 was induced in *PRKAA1*^*−/−*^ cells without enhanced transcription of *RELA* (data not shown), suggesting that the degradation of P65 is impaired in *PRKAA1*^*−/−*^ cells. The above-mentioned results indicate that AMPK inhibits NF-κB signaling in DAOY cells.

To determine AMPK inhibits the proliferation and metastasis of DAOY through NF-κB signaling, we performed a double-knockdown of both *PRKAA1* and *CCL5* in DAOY cells (Fig. 5H). Notably, *CCL5* depletion slowed down the accelerated cell proliferation induced by AMPK knockdown (Fig. 5I). More importantly, the enhanced migration and invasion ability of AMPK knockdown cells were significantly weakened by siCCL5 (Fig. 5J-M). Overall, these results demonstrated that AMPK attenuated DAOY cell growth and metastasis through inhibiting NF-κB pathway.

### NF-κB inhibitor and GDC-0449 synergistically suppresses MB cell growth

**Fig. 6:**
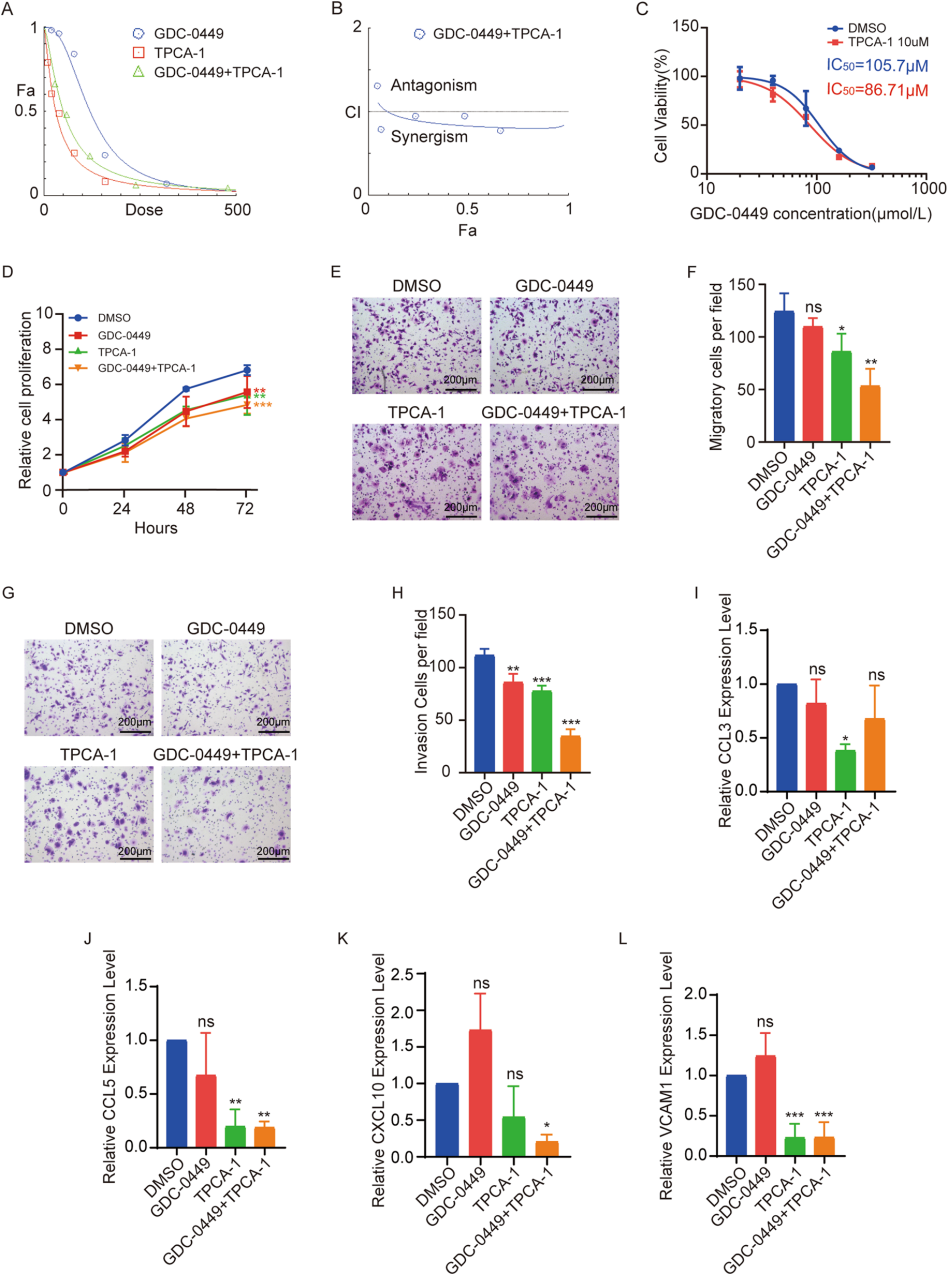
GDC-0449 and TPCA-1 synergistically suppresses DAOY growth and progression. **A** Dose–effect curve demonstrates the relationship between Fa and Dose. **B** The Combination index (CI) of GDC-0449 and TPCA-1. **C** Sensitization of TPCA-1. **D** Proliferation of *PRKAA1*^−/−^ DAOYs treated with GDC-0449 or TPCA-1 subjected to CCK-8 assay. Migration **E** and invasion **G** abilities of *PRKAA1*^−/−^ DAOYs treated with GDC-0449 or TPCA-1 evaluated by transwell assays with and without matrigel. (**F**) and (**H**) are quantification data of three independent experiments as in (**E**) and (**G**). RT-PCR detection of mRNAs of NF-κB downstream target genes *CCL3* (I), *CCL5* (J), *CXCL10* (K), and *VCAM1* (**L**) in *PRKAA1*^−/−^ DAOYs treated with drugs. Data in bar graphs are performed as mean ± SEM. * P < 0.05, ** P < 0.01, *** P < 0.001, ns not significant

Since NF-κB signaling contributes to the growth and metastasis of DAOY cells, targeting NF-κB may be employed for treatment in SHH-MB. Vismodegib (GDC-0449) is an FDA-approved SMO inhibitor targeting SHH-driven cancers. The CCK-8 assay revealed that GDC-0449 significantly decreased DAOY cell viability in a dose dependent manner. The IC_50_ value of it was determined to be 91.69 mM in *PRKAA1*^*−/−*^ DAOY cells (Additional file 4: Fig S4A). The IC_50_ value of NF-κB inhibitor TPCA-1 was 39.74 μM in *PRKAA1* knockout cells (Additional file 4: Fig S4B). We then performed analytics to determine whether GDC-0449 acted synergistically with TPCA-1 subjected to CCK-8 assay, *PRKAA1*^*−/−*^ DAOY cells were treated with GDC-0449 alone (0–320 μM), or TPCA-1 alone (0–160 μM), or a combination of both. To test for synergy, we validated the CCK-8 data using CompuSyn software based on Chou-Talalay quantitative method [21, 22]. The dose–effect curves showed the concentration-dependent cytotoxicity of the individual and combinational drugs at a constant ratio of 1:2 (160 μM TPCA-1 and 320 μM GDC-0449) (Fig. 6A). The combination index (CI) of TPCA-1 with GDC-0449 was less than 1, indicating the synergism of both drugs in reducing cell viability (Fig. 6B). In addition, we found that TPCA-1 increased the sensitivity of *PRKAA1*^*−/−*^ DAOY cells to GDC-0449 (Fig. 6C). Furthermore, GDC-0449 (40μM) worked synergistically with TPCA-1 (10μM) in the inhibition of proliferation (Fig. 6D), migration (Fig. 6E, F), and invasion (Fig. 6G, H) of *PRKAA1*^*−/−*^ DAOY cells. As expected, the transcripts of NF-κB pathway target genes (*CCL5, CXCL10,* and *VCAM1*) were reduced under the combinational drug treatment (Fig. 6I-L). Meanwhile, we performed double knockdown of *GLI1* and *CCL5* in *PRKAA1*^*−/−*^ cells simultaneously, and found that the double knockdown had additive inhibitory effect on cell growth (Additional file 5: Fig S5A) and migration (Additional file 5: Fig S5B, C) of *PRKAA1*^*−/−*^ DAOY cells. In brief, we showed that the combination of GDC-0449 and TPCA-1 had a synergically therapeutic effect on SHH-MB, revealing AMPK-NF-κB axis as a potential target for SHH-MB treatment.

## Discussion

Medulloblastoma (MB) is a highly malignant pediatric brain tumors (WHO grade IV), metastasis and recurrence are the main causes of death in MB patients [23]. SHH signaling plays an indispensable role during embryonic development, and aberrant activation of Shh pathway drives the origination of SHH subgroup MB, accounting for nearly one-third of MB cases [24]. Although the genetic divergence driving the primary tumorigenesis is commonly conserved at metastasis, the gene expression patterns of metastatic tumors remain distinct from non-metastatic primary tumors. Here, we report that lower expression of *PRKAA1* predicts poor prognosis of MB, and loss of AMPK promotes the growth, stemness and metastasis of SHH-MB cells. AMPK phosphorylates GLI1, then expedites the proteasome-mediated degradation of GLI1 by promoting the binding of GLI1 with its E3 ligase β-TrCP. This inhibitory activity of AMPK in Shh signaling partially explains the accelerated proliferation of SHH-MB cells lacking AMPKα.

NF-κB is one of the nuclear transcription factors function in a variety of biological processes, which exists in almost all types of mammalian cells. In most cancers, the activation of NF‐κB is enhanced due to increased stimuli of the NF‐κB pathway, such as increased TNFα and IL‐1 in the tumor microenvironment, which can accelerate cell proliferation, inhibit apoptosis, promote cell invasion and metastasis, and stimulate angiogenesis and elongation [25]. Genetic deletions affecting regulators of the NF-κB pathway, including NFKBIA and USP4, were identified in Group 4 MB [26]. Our study elucidated that AMPK attenuates further progress of SHH-MB by inhibiting NF-κB activation, slowing down cell proliferation, and arresting migration and invasion. Cerebrospinal fluid (CSF) is the window into the central nervous system, through which MB invade and disseminate to the spinal cord [27]. Although we could not collect enough cerebrospinal fluid from mice with intracranial tumor metastasis, recent clinical research analyzed the RNA-sequencing and high-resolution mass spectrometry data of CSF samples from patients with or without MB and reported differentially expressed genes enriched in TNF-α signaling via NF-κB, which supports our results strongly [27]. Overall, our results demonstrate that AMPK attenuates the growth and metastasis of SHH-MB by inhibiting NF-κB activity.

Elucidating the mechanism of MB metastasis provides the opportunity to prevent its metastasis and to reduce the mortality of MB patients. In recent years, FDA-approved Smo antagonists Vismodegib and Sonidegib were available for SHH-driven tumors, but drug resistance and relapse have also appeared in clinical use [28]. Metformin and A769662 activate AMPK, then inhibit GLI1 activity to restrain MB cells growth in vitro and in vivo, thereby sensitizing SHH-MB to Vismodegib and overcoming Vismodegib-resistance [20]. However, AMPK agonists may not be effective in metastatic MB due to the low expression of AMPKα. So we sought to explore the novel molecular targets of MB therapy at the downstream of AMPK. AMPK-NF-κB axis functions in the metastasis of SHH-MB. There have been thousands of molecules described as capable of interfering with the NF-κB signaling directly or indirectly [25]. TCPA-1 is one of them interfering with the activation of NF-kB by selectively inhibiting IKK-2. It has been used in tumor therapy trials [29, 30]. Based on these considerations, we combined SHH and NF-κB inhibitors Vismodegib and TPCA-1 to treat SHH-MB lacking AMPKα. Our results represented that these two drugs synergistically restrain the growth, migration, and invasion of SHH-MB cells in vitro, suggesting that therapies might be efficacious for SHH-MB at metastasis and recurrence. It is necessary to conduct more research in the future to clarify the importance of activating AMPK and inhibiting GLI1 and NF-κB pathways for the prevention of metastasis in an in vivo model to determine whether they have clinical translational value.

## Conclusions

**Fig. 7:**
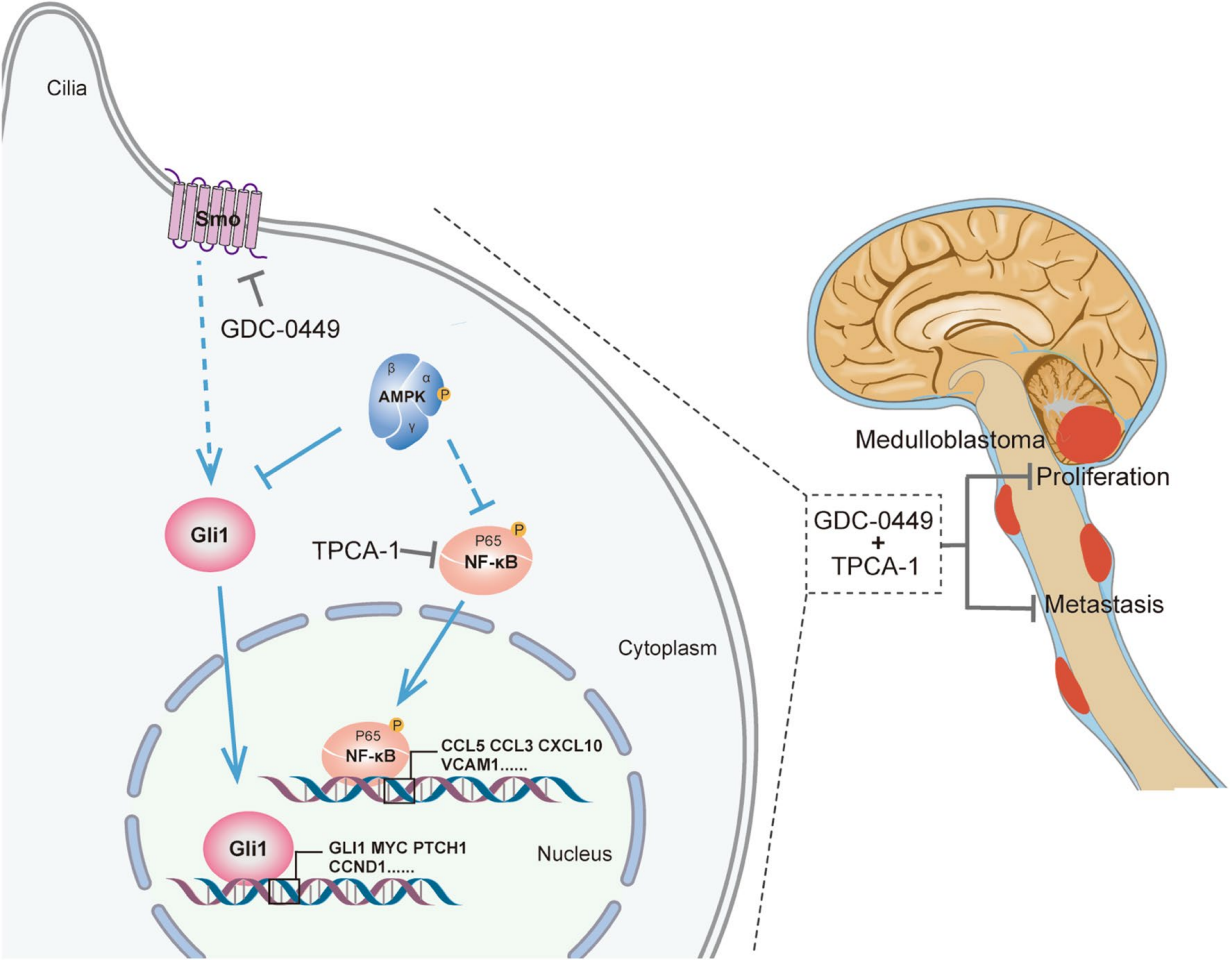
Diagram representation of AMPK function as a tumor suppressor in MB development. AMPK functions through Gli1 and NF-κB in MB development. GDC-0449 and TPCA-1 synergistically restrain MB progression

This study demonstrated that AMPK functions as a tumor suppressor in MB progression through two signaling pathways, SHH-GLI1 and NF-κB (Fig. 7). NF-κB inhibitor and Shh inhibitor synergistically suppress MB cell growth, revealing the AMPK-NF-κB axis as a potential target for molecular therapy of SHH-MB. The anti-cancer effect of a combination of Vismodegib and TPCA-1 was superior to Vismodegib alone in MB targeted therapy (Fig. 7). Future studies are required to ascertain whether these two drugs have synergistically therapeutic effects in vivo.

## Materials and methods

### Cells, plasmids, and siRNAs

Human cell lines were purchased from ATCC and FuHeng, and maintained with 1% penicillin and streptomycin (Gibco Life Technologies) at 37 °C with 5% CO_2_. DAOY cells (ATCC Cat# HTB-186, RRID:CVCL 1155) were cultured in DMEM medium supplemented with 10% fetal bovine serum (FBS, Gibco), 1 × glutamine (Gibco), and 1 mM sodium pyruvate (Gibco). D283-Med cells (ATCC Cat# HTB-185, RRID:CVCL_1155) were cultured in MEM medium supplemented with 10% FBS. Full-length human *PRKAA1* cDNA was generated by PCR and cloned into the pRK5 vector (RRID:Addgene_32693). siRNAs specific for the human *PRKAA1, GLI1* and *CCL5* were purchased from GenePharma (Shanghai, China).

### Immunohistochemical staining

Medulloblastoma tissues obtained from Children’s hospital of Nanjing Medical University (Nanjing, China) were carried out to detect the phosphorylation of AMPKα by immunohistochemistry. Mouse heterograft tumor tissues were detected Ki67 to measure the growth of heterograft tumors. And standard procedures for immunohistochemical staining were followed with rabbit two-step detection kit (ZSGB-BIO). The primary antibodies were rabbit anti-phospho-AMPKα (Cell Signaling Technology; 1:100), rabbit anti-Ki67 (Abcam; 1:100).

### Immunoblotting

Transfected cells or grinded tumor tissues were lysed in modified RIPA buffer (50 mM Tris–HCl, pH 7.4, 150 mM NaCl, 1% vol/vol NP-40, 1% n-Dodecyl β-D-maltoside, 0.25% wt/vol sodium deoxycholate, 1 mM DTT, and 1 × Roche complete Protease Inhibitor Cocktail) for 1 h at 4 ℃. The lysate was clarified by centrifugation for 20 min at 14,000 × g. The protein concentration was determined using a bicinchoninic acid assay and equal amounts of total protein from each of the samples was supplemented with 5 × SDS loading buffer, incubated at 95 ℃ for 5 min, subjected to SDS-PAGE, followed by western blot analysis. The following antibodies were used: rabbit anti-β-actin (Affinity; 1:5000), rabbit anti- AMPKα (Cell Signaling Technology; 1:1000), rabbit anti-Phospho-AMPKα (Cell Signaling Technology; 1:1000), mouse anti-E-cadherin (Cell Signaling Technology; 1:1000), rabbit anti-N-cadherin (Elabscience; 1:1000), rabbit anti-MMP3 (Proteintech; 1:1000), rabbit anti-GLI1 (Cell Signaling Technology; 1:1000), rabbit anti-GLI2 (NOVAS; 1:1000), goat anti-GLI3 (R&D; 1:1000), mouse anti-IκBα (Cell Signaling Technology; 1:1000), rabbit anti-NF-κB p65 (Cell Signaling Technology; 1:1000) and rabbit anti-Phospho-NF-κB p65 (Cell Signaling Technology; 1:1000).

### Cell Count Kit-8 (CCK-8) assays

Treated DAOY and D283-Med cells were separately seeded in 96-well plates at a density of 0.4 × 10^4^ cells/well and 1 × 10^4^ cells/well. After adhesion, the cell viability was obtained by the CCK-8 kit for 72 h. The following formulae were used for calculations:

Cell viability (%) = ([OD (experiment) – OD (blank)])/ ([OD (control) – OD (blank)]) × 100%

### EdU incorporation assays

Transfected cells were seeded at 5 × 10^4^ cells/well in 24-well plates containing round coverslips and maintained in medium overnight. Cell proliferation was further evaluated through measuring the incorporation of EdU with EdU Cell Proliferation Assay Kit (RiboBio). Images were captured by using a fluorescence microscope, and the proliferating cells in five different fields were counted.

### Colony formation assays

For the colony formation assay, transfected cells were plated in triplicate at 200 cells per well in 6-well plates and cultured for 2 weeks. The cells were fixed with 4% paraformaldehyde for 15 min at 4 ℃, and then stained with 0.1% crystal violet for 30 min, colonies with more than 50 cells were counted.

### Flow cytometric analysis for cell cycle

Cells were inoculated in 6-well plates and transfected with siRNA for 48 h, then these cells were collected and fixed with pre-cooled 75% ethanol. Then cell cycle distribution was determined by the flow cytometer (BD Biosciences).

### Tumorsphere assays

Tumorsphere formation assays were measured by in *vitro* limiting dilution assay, as previously reported [31, 32]. Briefly, decreasing numbers of cells per well (50, 20, 10, 5 and 1) were plated into Ultra-Low Attachment 96-wells plates (Costar®) in Neural basal medium (Gibco) supplemented with 100 × penicillin and streptomycin (Gibco Life Technologies), 100 × Glutamine (Gibco), 100 × D-( +)-Glucose solution (Sigma), 100 × Insulin-Transferrin-Selenium (Gibco), 50 × B27 Supplement (Gibco), N-Acetyl Cysteine (16ug/ml), EGF (20 ng/ml) and FGF (20 ng/ml). Spheres with a diameter equal or higher than 40 μm were deemed tumourspheres. The presence and number of tumorspheres in each well were recorded seven days after plating. Extreme limiting dilution analysis was performed using the software available at [31, 33].

### CRISPR-Cas9 genome editing

Genome editing was achieved using the CRISPR-Cas9 technique in DAOYs. Briefly, a single guide RNA (sgRNA) targeted the eighth and tenth exon of human *PRKAA1* was designed and cloned into pX330 vector (RRID: Addgene_101733). The sgRNA sequences are:

5′- CACCGAACTATATGATGGATCCTC -3′;

5′- CACCGCAACTATCGATCTTGCCAA -3′.

Cells were co-transfected with CRISPR/Cas9 plasmids and empty plasmids containing puromycin resistance genes. 48 h after transfection, transfected cells were selected using Puromycin (3 μg/ml) for 7 days, followed by another 4 days without selection for expansion. Then the survived DAOYs were seeded at 1 cell per well in 96-well plates for single cell colonies formation and screened by genotyping and Western Blot.

### In vivo xenograft tumor model

6 weeks female BALB/c nude mice were purchased from Nanjing Medical University Experimental Animal Center. Nude mice were subcutaneously inoculated with 5 × 10^6^ *PRKAA1*^+*/*+^ or *PRKAA1*^*−/−*^ DAOY cells in double side axillae. The tumor sizes were measured every week. The tumor volume was calculated as follows: volume (mm^3^) = length (mm) × width (mm)^2^/2. 8 weeks after xenograft inoculation, mice were euthanized and their tumor nodules were excised, photographed, weighed and harvested.

### Intracranial brain tumor xenografts

NOD-SCID mice were anesthetized, and an incision was made to expose the skull. A hole was created in the calvarium above the right cerebellar hemisphere, where 2 mm lateral (right) to the sagittal suture, and 2 mm posterior of the lambdoid suture, using a microdrill. Orthotopic MB xenografts were performed by injection of *PRKAA1*^+*/*+^ DAOY-Luc and *PRKAA1*^*−/−*^ DAOY-Luc cells severally, that had been previously infected with lentiviral particles to stably express luciferase gene, into the cerebellum of five NOD-SCID mice. Bioluminescence imaging was performed weekly using IVIS Spectrum (Perkin Elmer). Tumor growth was evaluated by quantifying the bioluminescence signals using the integrated fluxes of photons within each area of interest using the Living Images Software Package 4.5 (Perkin Elmer). All mice were euthanized when the mice showed symptoms of metastasis.

### Movement of living cells

Cells were seeded in 60-mm plate at 30% density for 12 h and then treated with 2 μg/ml mitomycin C for 24 h. After, cells were cultured with fresh culture medium and recorded at one image every 5 min with Celldiscoverer 7 automatic live cell imaging system (ZEISS) for 20 h. The cells motion trajectory image and data were obtained using ImageJ (RRID:SCR_003070).

### Cytoskeleton staining

Transfected cells were seeded on glass coverslips for 24 h and fixed with 4% PFA for 20 min at 4℃, then the cells were permeabilized with 0.5% TritonX-100 for 10 min at room temperature. Fluorescently labeled phalloidin working solution was added a well and incubated at room temperature for 30 min for staining and stained with DAPI. The cell cytoskeleton images were acquired with laser scanning confocal microscope.

### In vitro migration and invasion assays

The 24-well plate with 8 µm pore polycarbonate membrane inserts (Millipore) was used to analyze the migratory abilities of tumor cells and then the membrane was coated with 60μL diluted Matrigel (1:30; Corning) to detect cells’ invasion. After adding 600μL 10% FBS medium into the lower chambers, 2.5 × 10^4^ transfected cells in 300μL serum-free medium were seeded into the insert for incubation at 37 °C in 5% (v/v) CO_2_ incubator for 16 h. Then, the cells migrating to the lower surface of the membrane insert were stained with the crystal violet (Beyotime) and quantified by counting five randomly chosen microscopic fields.

### RNA sequencing

Total RNA was isolated with RNAiso Plus reagent (TaKaRa) from DAOY cells transfected with siNC or siPRKAA1 for 48 h, then purified to meet the following requirements were used in subsequent experiments: RNA integrity number (RIN) > 7.0 and a 28S:18S ratio > 1.8. The triplicate samples of both assays were constructed an independent library, and sequenced on an Illumina Novaseq 6000 sequencer by CapitalBio Technology (Beijing, China). The sequencing raw data was uploaded to the GEO dataset (GSE218076).

### Reverse transcription (RT) and real-time PCR

Total RNAs were isolated from cultured cells and reverse transcribed using HiScript II Q RT SuperMix (Vazyme). Quantitative real-time PCR (qPCR) was carried out using AceQ qPCR SYBR Green Master Mix (Vazyme). Each measurement was repeated three times, and each sample was analyzed in triplicate with hypoxanthine phosphoribosyl transferase (HPRT) as an internal control. The qPCR primers are listed:

Human *PRKAA1*: Forward: 5′- CAACTATCGATCTTGCCAAAGG -3′

Reverse: 5′- AACAGGAGAAGAGTCAAGTGAG -3′

Human *CCL3*: Forward: 5′- AGGACACGGGCAGCAGACAG -3′

Reverse: 5′- GGACAGCAAGGGCAGCAGTG -3′

Human *CCL5*: Forward: 5′- CAGCAGTCGTCCACAGGTCAAG -3′

Reverse: 5′- TTTCTTCTCTGGGTTGGCACACAC -3′

Human *CXCL10*: Forward: 5′- CTCTCTCTAGAACTGTACGCTG -3′

Reverse: 5′- ATTCAGACATCTCTTCTCACCC -3′

Human *VCAM1*: Forward: 5′- CAGGCTGGAGATAGACTTACTG -3′

Reverse: 5′- CCTCAATGACAGGAGTAAAGGT -3′

### Combination index with simultaneous treatment of Vismodegib and TPCA-1

To understand the Combination Index (CI), cells were treated with Vismodegib (GDC-0449) alone, or TPCA-1 alone, or a combination of both in the following concentrations (0, 1/4 IC_50_, 1/2 IC_50_, IC_50_, 2 IC_50_, and 4 IC_50_). To test for synergy, and then the CCK8 data was validated using CompuSyn software based on Chou-Talalay quantitative method. The dose–effect curve was simulated and CI was calculated at a constant ratio of 1:2.

### Analysis of published datasets

Human medulloblastoma expression dataset from GSE85217 and GSE124814 was used. Correlations between different gene were determined by Spearman correlation analysis. Overall survival curves were analyzed with Kaplan–Meier plotter (https://hgserver1.amc.nl/cgi-bin/r2/main.cgi). The best cutoff value was auto selected in the analysis. The hazard ratio with 95% confidence interval and log rank P value were calculate, and significance was set at P < 0.05.

### Statistical analysis

Statistical analyses were performed with GraphPad Prism 8.0 (RRID:SCR_002798). Each measurement was repeated at least three times. Comparisons between indicated groups were performed using independent-samples t-test. P values < 0.05 were considered statistically significant. *P < 0.05, **P < 0.01, and ***P < 0.001. n.s. not significant.

## Supplementary Information

**Additional file 1: Figure S1.** Knockdown of AMPKα promotes MB cell lines proliferation. (A) Western blot detection of AMPKα and P- AMPKα in DAOYs transfected with siRNAs. (B) CCK-8 assays for DAOYs expressing siRNAs. (C) Representative fluorescent images and (D) percentage quantification of EdU incorporation assays as in (C). (E) Colony counting of DAOYs expressing siRNAs and the quantification data of three independent experiments (E). (G) Cell cycle detection by flow cytometry and the quantification (H). (I) RT-PCR detecting PRKAA1 knockdown efficiency in D283-Med cells. (J) Proliferation of D283-Meds expressing siRNA was assessed by CCK-8. Quantification data from three independent experiments are presented as mean ± SEM. * P < 0.05, ** P < 0.01, *** P < 0.001

**Additional file 2: Figure S2.** Knockout of AMPKα promotes the growth of DAOY-derived subcutaneous xenografts. (A) Pattern graph of PRKAA1-/- DAOY cells. (B) Images of subcutaneous xenograft models of nude mice. (C) Tumor volume was weekly measured for each mouse. (D) Tumor weight was measured 8 weeks after xenograft inoculation. (E) IHC staining of Ki67 of subcutaneous tumors (40x)

**Additional file 3: Figure S3.** Knockdown of AMPKα promotes MB cell lines migration and invasion. (A) Cytoskeleton staining of DAOYs transfected with PRKAA1 siRNA. Transwell assays detecting the migration and invasion of DAOY cells expressing siRNA. Representative images of DAOY cell migration (B) and invasion (D). Quantification of numbers of migratory cells (C) and invading cells (E) per field. (F) Transwell migration assay of D283-Meds expressing siRNAs and the quantification (G). Quantification data from three independent experiments are presented as mean ± SEM, *** P < 0.001. Western blot detection of EMT markers in subcutaneous xenografts (H) and DAOYs transfected with siRNAs (I)

**Additional file 4: Figure S4.** GDC-0449 and TPCA-1 inhibit the growth of PRKAA1-/- DAOY cells dose dependently. The IC50 of GDC-0449 (A) and TPCA-1 (B) against PRKAA1-/- DAOY cells

**Additional file 5: Figure S5.** Double knockdown of GLI1 and CCL5 collaboratively inhibits the proliferation and invasion of PRKAA1-/- DAOY cells. (A) Proliferation of PRKAA1-/- DAOYs transfected with siGLI1 and siCCL5 alone or together were assessed by CCK-8. (B) Transwell invasion assay of PRKAA1-/- DAOYs transfected with siRNAs and its quantification data (C) performed the average invasion ± SEM from three independent experiments. (D) RT-PCR detection of GLI1 and CCL5 mRNAs in PRKAA1-/-DAOYs transfected with siRNAs. * P < 0.05, *** P < 0.001

## Data Availability

All data generated or analyzed during this study are included in this published article and its additional files.

## Abbreviations

MB: Medulloblastoma
Shh: Sonic Hedgehog
SHH-MB: SHH subgroup of Medulloblastoma
AMPK: AMP-dependent protein kinase
CNS: Central nervous system
WNT: Wingless
Ptch-1: Patched-1
Smo: Smoothened
TCGA: The Cancer Genome Atlas
SiRNA: Small interfering RNA
Luc: Luciferase
BLI: Bioluminescent imaging
F-Actin: Actin filaments
EMT: Epithelial-mesenchymal transition
GSEA: Gene set enrichment analysis
CI: Combination index
CSF: Cerebrospinal fluid
