# Supplementary figures and images for: AMPK attenuates SHH subgroup medulloblastoma growth and metastasis by inhibiting NF-κB activation
Source: Cell Biosci. 2023 Jan 22;13:15. doi: 10.1186/s13578-023-00963-2 (PMC9867863; doi:10.1186/s13578-023-00963-2)

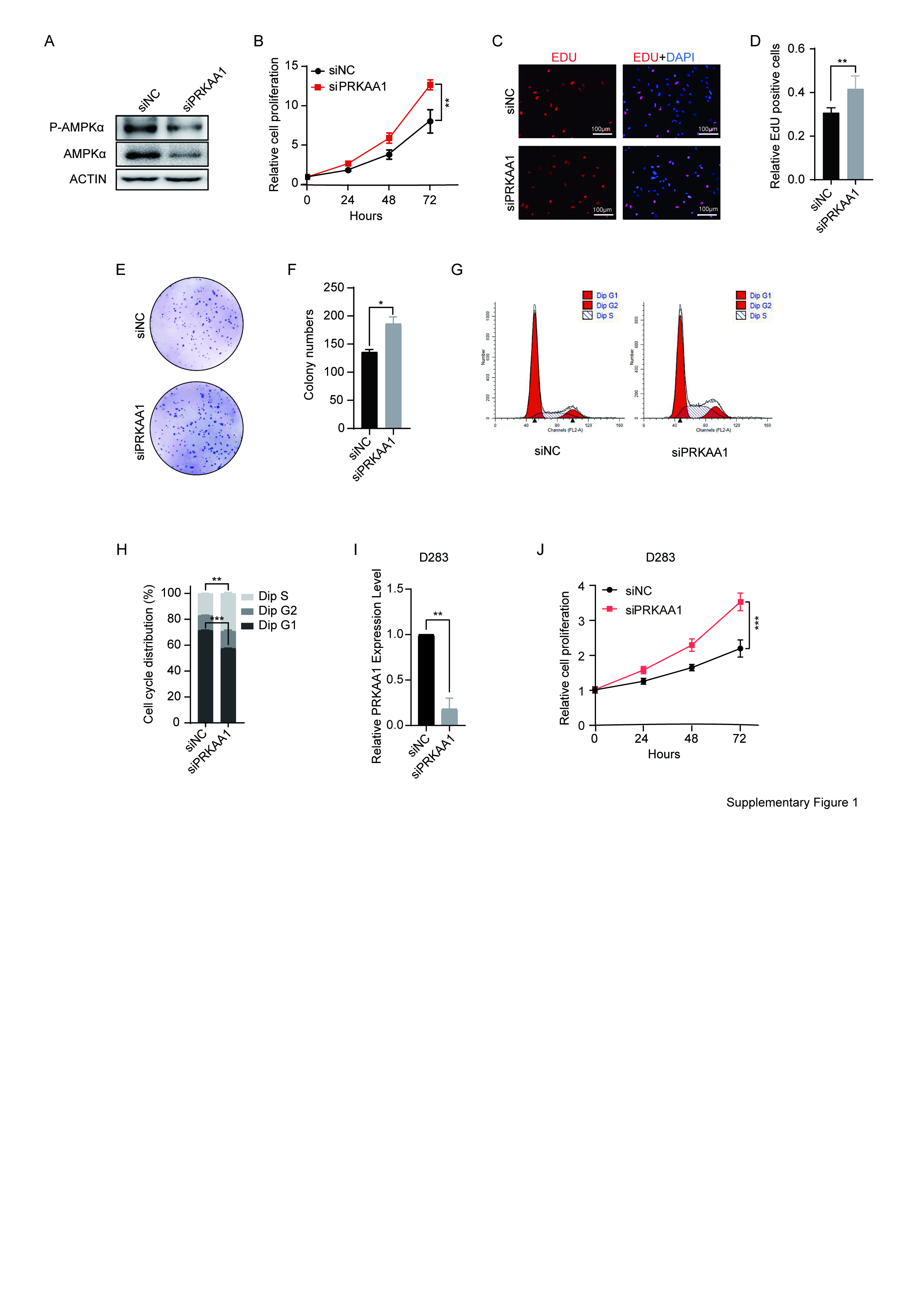

Supplement: Supplementary file 1 — Additional file 1: Figure S1. Knockdown of AMPKα promotes MB cell lines proliferation. (A) Western blot detection of AMPKα and P- AMPKα in DAOYs transfected with siRNAs. (B) CCK-8 assays for DAOYs expressing siRNAs. (C) Representative fluorescent images and (D) percentage quantification of EdU incorporation assays as in (C). (E) Colony counting of DAOYs expressing siRNAs and the quantification data of three independent experiments (E). (G) Cell cycle detection by flow cytometry and the quantification (H). (I) RT-PCR detecting PRKAA1 knockdown efficiency in D283-Med cells. (J) Proliferation of D283-Meds expressing siRNA was assessed by CCK-8. Quantification data from three independent experiments are presented as mean ± SEM. * P < 0.05, ** P < 0.01, *** P < 0.001 [file 13578_2023_963_MOESM1_ESM.tif]

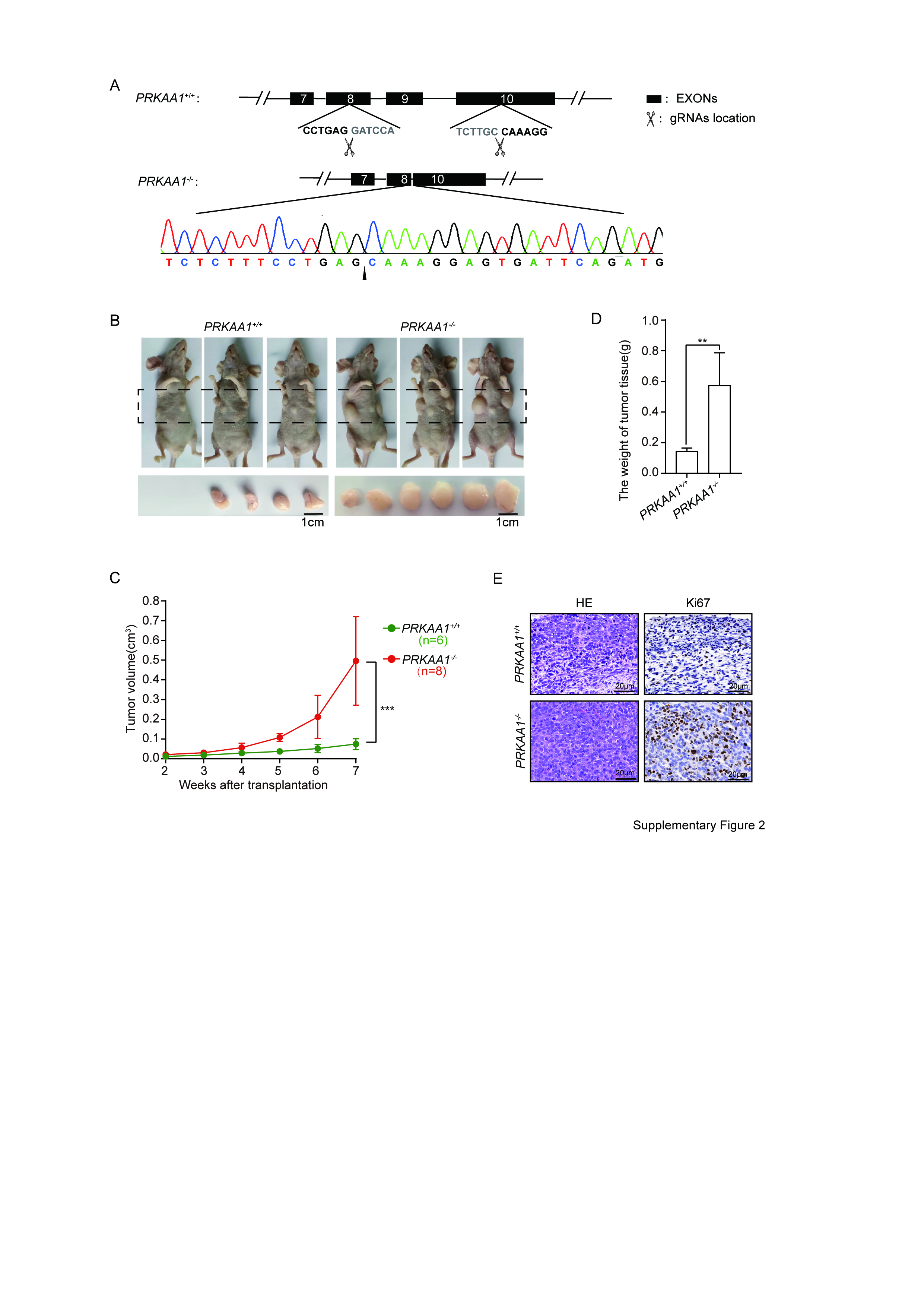

Supplement: Supplementary file 2 — Additional file 2: Figure S2. Knockout of AMPKα promotes the growth of DAOY-derived subcutaneous xenografts. (A) Pattern graph of PRKAA1-/- DAOY cells. (B) Images of subcutaneous xenograft models of nude mice. (C) Tumor volume was weekly measured for each mouse. (D) Tumor weight was measured 8 weeks after xenograft inoculation. (E) IHC staining of Ki67 of subcutaneous tumors (40x) [file 13578_2023_963_MOESM2_ESM.tif]

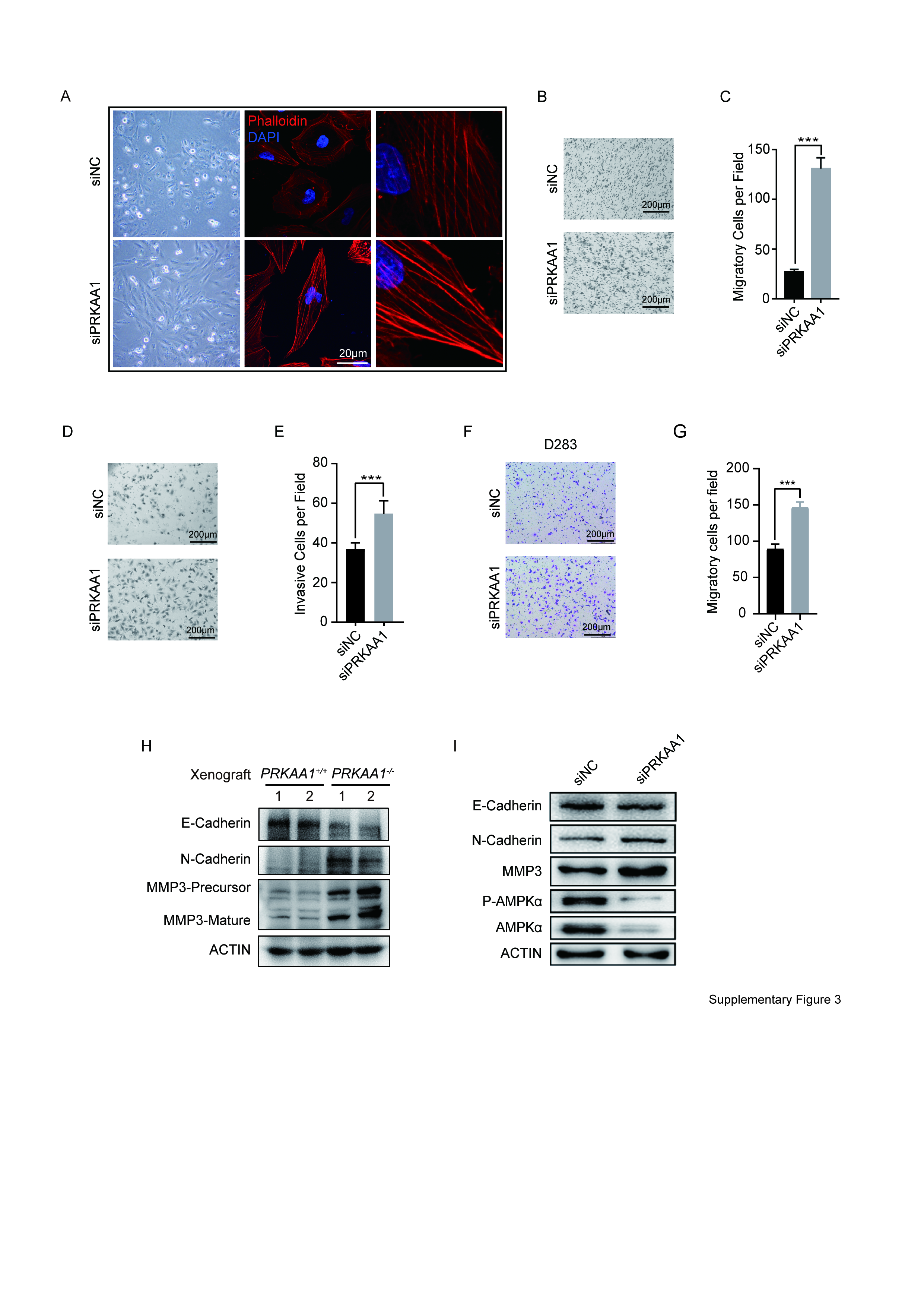

Supplement: Supplementary file 3 — Additional file 3: Figure S3. Knockdown of AMPKα promotes MB cell lines migration and invasion. (A) Cytoskeleton staining of DAOYs transfected with PRKAA1 siRNA. Transwell assays detecting the migration and invasion of DAOY cells expressing siRNA. Representative images of DAOY cell migration (B) and invasion (D). Quantification of numbers of migratory cells (C) and invading cells (E) per field. (F) Transwell migration assay of D283-Meds expressing siRNAs and the quantification (G). Quantification data from three independent experiments are presented as mean ± SEM, *** P < 0.001. Western blot detection of EMT markers in subcutaneous xenografts (H) and DAOYs transfected with siRNAs (I) [file 13578_2023_963_MOESM3_ESM.tif]

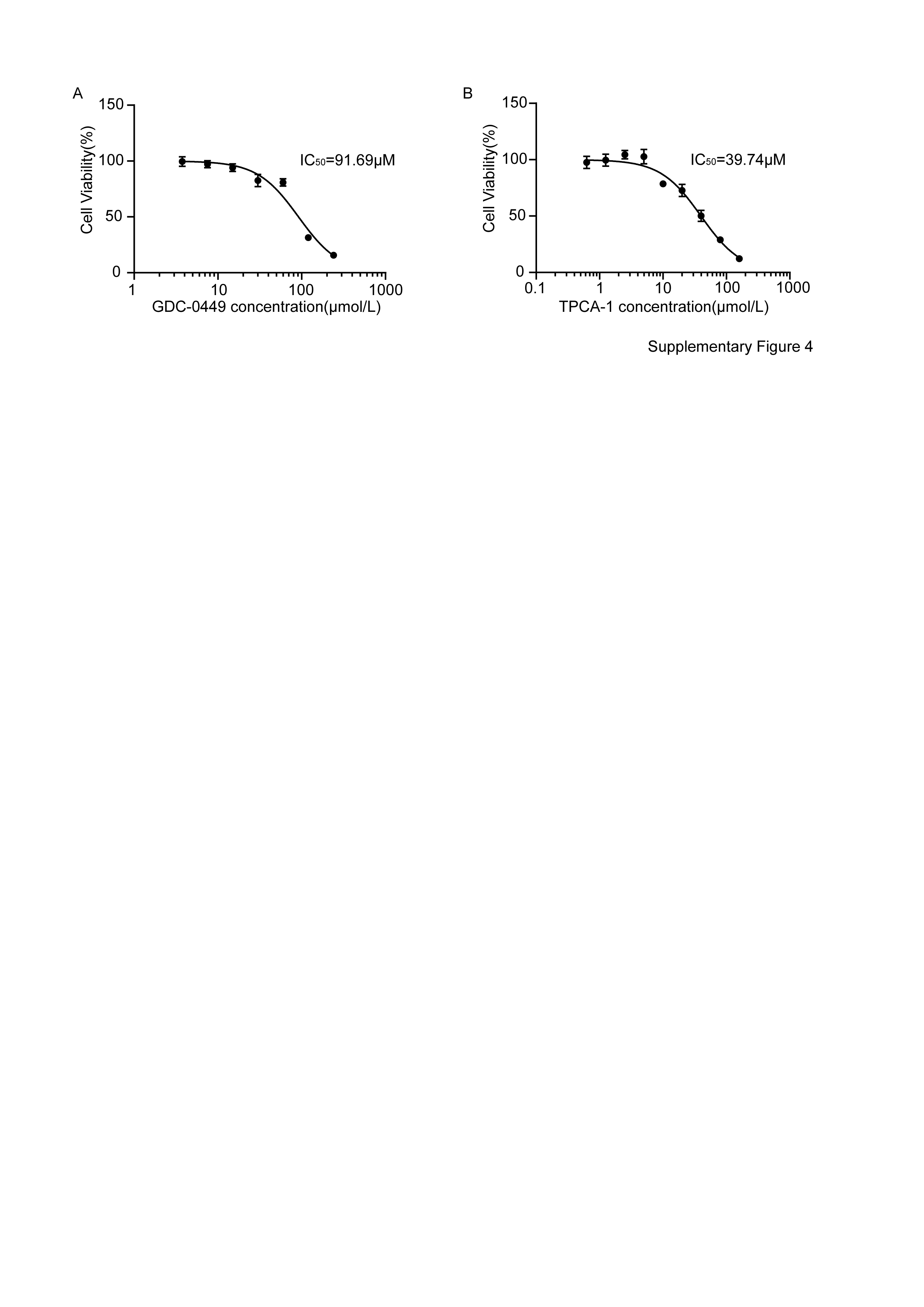

Supplement: Supplementary file 4 — Additional file 4: Figure S4. GDC-0449 and TPCA-1 inhibit the growth of PRKAA1-/- DAOY cells dose dependently. The IC50 of GDC-0449 (A) and TPCA-1 (B) against PRKAA1-/- DAOY cells [file 13578_2023_963_MOESM4_ESM.tif]

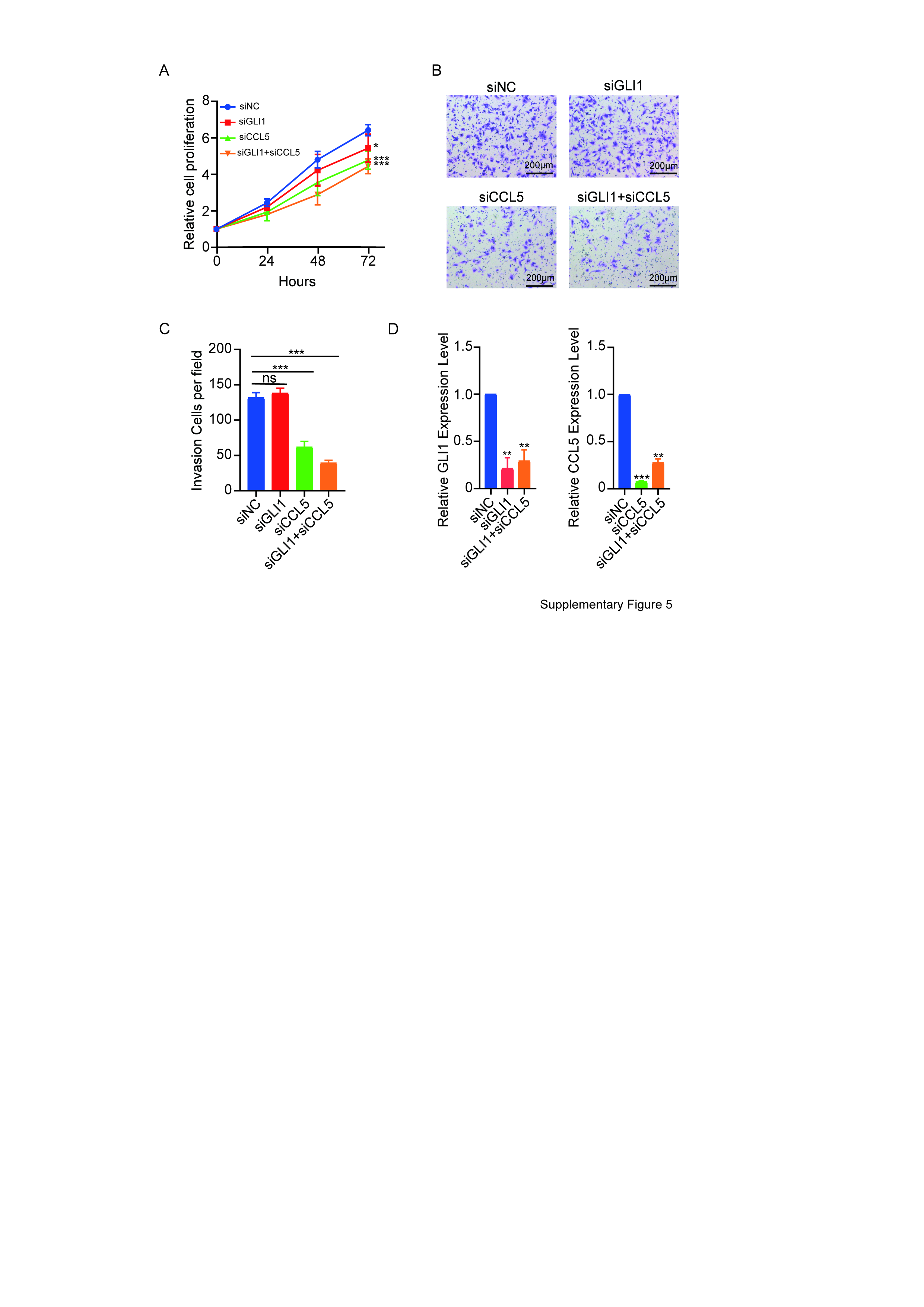

Supplement: Supplementary file 5 — Additional file 5: Figure S5. Double knockdown of GLI1 and CCL5 collaboratively inhibits the proliferation and invasion of PRKAA1-/- DAOY cells. (A) Proliferation of PRKAA1-/- DAOYs transfected with siGLI1 and siCCL5 alone or together were assessed by CCK-8. (B) Transwell invasion assay of PRKAA1-/- DAOYs transfected with siRNAs and its quantification data (C) performed the average invasion ± SEM from three independent experiments. (D) RT-PCR detection of GLI1 and CCL5 mRNAs in PRKAA1-/-DAOYs transfected with siRNAs. * P < 0.05, *** P < 0.001 [file 13578_2023_963_MOESM5_ESM.tif]
